# Facing the COVID‐19 pandemic: An Italian feasibility study of a mixed in‐person/telerehabilitation intervention for cancer patients

**DOI:** 10.1002/cam4.70022

**Published:** 2024-08-02

**Authors:** Monica Denti, Alessia Pecorari, Monia A. Accogli, Stefania Costi, Carlotta Mainini, Martina Pellegrini, Chiara Boni, Bressi Barbara, Luca Braglia, Stefania Fugazzaro

**Affiliations:** ^1^ Physical Medicine and Rehabilitation Unit Azienda Unità Sanitaria Locale—IRCCS di Reggio Emilia Reggio Emilia Italy; ^2^ Unit of Dentistry and Oral‐Maxillo‐Facial Surgery, Department of Surgery, Medicine, Dentistry and Morphological Sciences, with Transplant Surgery, Oncology and Regenerative Medicine Relevance (CHIMOMO) University of Modena and Reggio Emilia Modena Italy; ^3^ Department of Surgery, Medicine, Dentistry and Morphological Sciences University of Modena and Reggio Emilia Modena Italy; ^4^ Clinical Trials Center, Azienda USL‐IRCSS di Reggio Emilia Reggio Emilia Italy

**Keywords:** cancer patients, exercise, feasibility, physical activity, physiotherapy, telemonitoring, telerehabilitation

## Abstract

**Introduction:**

The COVID‐19 pandemic impacted healthcare organizations, necessitating a rapid transition from in‐person to virtual care.

Our study explored the feasibility of a mixed in‐person/telerehabilitation intervention for cancer patients and its effects on cancer‐related fatigue (CRF), quality of life (QoL), physical function, patient satisfaction, and perceived usefulness.

**Methods:**

TRACE 2020 is an observational prospective study that enrolled adult cancer patients, between January 2021 and March 2023, who were eligible for a rehabilitation program to be provided also in telerehabilitation.

Patients were assessed at baseline and after the rehabilitation program. Adherence to sessions, reasons for non‐adherence and adverse events were collected.

**Results:**

Of the 23 patients enrolled, 87% received a mixed intervention, with an average of 60% in‐person sessions and 40% telerehabilitation sessions. Adherence was very high (91%). Evaluation scales showed an improvement in CRF, QoL, and lower limb strength and a relevant increase in patients' level of physical activity (PA). Most patients reported good satisfaction; the few criticisms mainly concerned difficulties in connectivity, lack of physical contact and difficulties in understanding how to perform exercises during telerehabilitation sessions. The physiotherapist underlined the usefulness of the innovative approach and suggested ways to facilitate future implementation.

**Conclusion:**

A mixed intervention including telerehabilitation is feasible and accepted by cancer patients. It may have a positive effect on their CRF, QoL, and level of PA and render patient care more flexible. The findings suggest what characteristics the target population for telerehabilitation should have, in order to integrate telerehabilitation in standard care for cancer patients.

## Graphical abstract

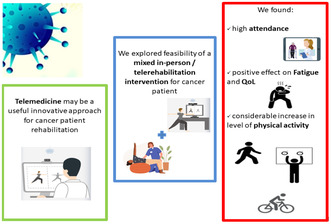

## 1 INTRODUCTION

Since it was declared a global pandemic in March 2020, coronavirus SARS‐CoV‐2 had a deep impact not only on people's health worldwide but also on healthcare organizations and on patient care in the rehabilitation context.

According to the Guidelines issued by the Italian Ministry of Health,^1^ in the period March–April 2020, outpatient activities were greatly reduced, including those in rehabilitation, and only non‐deferrable services were guaranteed; limitations were maintained until the first months of 2021 regarding.

The coronavirus disease 2019 (COVID‐19) pandemic required a rapid transition from in‐person rehabilitation to telephone‐ or videoconferencing‐based virtual care,^2^ also prompted by WHO indications.^3^

The application of telemedicine to rehabilitation was proposed in the early 2000s as a way to increase access to treatment and supportive care for populations with disabilities, focusing on the potential savings in time and cost.^4^ In Italy, the number of patients assisted in telerehabilitation has increased since 2007, although primarily when participating in research projects, not in clinical standard care.^5^

Before the COVID‐19 pandemic, telemedicine had been investigated mainly in neurologic and cardiac rehabilitation programes.^4^

Some studies, published since 2016, reported this approach also in cancer patients, finding it promising, in particular to reduce fatigue, improve quality of life (QoL), and patient‐reported adherence and satisfaction.^6^, ^7^

Galiano‐Castillo et al explored the benefits of an internet‐based supervision on breast cancer patients after chemotherapy and found positive effects on global health status, strength, pain, cognitive functioning, and fatigue.^8^

Villaron et al reported in 2018 that a weekly SMS text message for exercise promotion associated with a recommendation booklet and a pedometer could improve self‐reported fatigue and QoL in a mixed cancer population, while no change was detected in level of exercise.^6^

Gehring et al conducted a pilot randomized controlled trial (RCT) on patients with stable grade II and III gliomas exposed to a 6‐months home‐based, remotely guided exercise intervention (three sessions per week). Participants improved cardiorespiratory fitness, but the sample was small.^7^

Buneviciene et al reported in 2021 that interventions based on the use of mobile phones, patient monitoring devices, and other wireless devices were promising for improving the Health‐Related Quality of Life (HRQoL) of patients with cancer, and their systematic review found good evidence for improving physical activity/fitness, with positive effect in HRQoL.

They explored the promotion of physical activity and fitness intervention in a heterogeneous population of cancer patients (mostly breast cancer, but also lung, prostate, and colorectal cancer), during anticancer treatments.^9^

The duration of the intervention varied on average from 6 to 12 weeks, with usually weekly monitoring, via device or contact with healthcare professionals (HCP).^9^

At the onset of the pandemic, the use of telerehabilitation for cancer patients as well took a step forward, as the risk of severe illness after contracting SARS‐CoV‐2 were higher in this population, principally due to their frailty, advanced age, and the presence of comorbidities.^10^, ^11^

In 2021, Chang and Asher recommended telemedicine use in cancer rehabilitation, including exercise and lifestyle education, with the advantage of enhanced accessibility by overcoming known barriers to participation in in‐person supervised exercise, reducing cost, and travel burden.^12^

During the COVID‐19 pandemic, it was necessary to introduce telerehabilitation into our context as well to allow the continuation of rehabilitation care of cancer patients. At the same time, we considered it useful to evaluate the feasibility and safety of an innovative mixed approach in cancer rehabilitation, not yet well investigated. In fact, studies published till 2020 focused on remote rehabilitation programs,^13^, ^14^, ^15^, ^16^ while the use of remote cancer care or monitoring, associated to an in‐person rehabilitation intervention, was not sufficiently explored.

We therefore designed the Telerehabiliation after COVID Emergency (TRACE) 2020 project with the aim of evaluating the feasibility of telerehabilitation by analyzing patients' adherence to a mixed in‐person/remote rehabilitation intervention. The secondary objective was to investigate the effects of the intervention on patient‐reported outcomes, with a focus on cancer‐related‐fatigue, QoL, physical function, and patient satisfaction and perceived usefulness.

## 2 MATERIALS AND METHODS

TRACE 2020 is a monocentric observational prospective study conducted at the Santa Maria Nuova Hospital (ASMN) in Reggio Emilia between January 1, 2021 and June 30, 2023. Santa Maria Nuova Hospital is a Comprehensive Cancer Care and a multi‐professional team including physiatrists, physiotherapists, and occupational therapists, offers cancer rehabilitation to outpatients and inpatients in clinical pathways and in research projects. TRACE 2020 did not leverage a clinical program at our hospital, but it was an innovative project.

The study was approved by the local Ethics Committee of the AUSL di Reggio Emilia on December 18, 2020 (No. 2020/0149587, Chairperson Dr. Sebastiano Calandra Buonaura).

The manuscript was written according to STROBE guidelines for reporting data of observational studies.^17^

### 2.1 Population

Participants were cancer patients needing outpatient rehabilitation. We included patients with all types of cancer, stage I, II, III, and IV, after cancer surgery or during active therapy (chemotherapy and/or monoclonal antibodies and/or radiotherapy, etc.) or after oncological treatment.

Inclusion and exclusion criteria are shown in Table 1.

**TABLE 1:** Inclusion and exclusion criteria.

Inclusion criteria	Exclusion criteria
Age ≥18 years	Need for physiotherapist contact and presence during the outpatient session
Need of an individual personalized rehabilitation program, to be provided partially via telerehabilitation	Language barrier
Internet access and technological device suitable for telemedicine	Presence of sensorial, cognitive, or other deficits that prevent collaboration in rehabilitation
Informed consent	Patients with pathological fractures or unstable bone metastases
	Patients with absolute contraindications for exercise according to International Guidelines^18^

We enrolled patients from January 1, 2021 to March 31, 2023.

Patients were screened by physiatrists with an in‐person visit.

### 2.2 Intervention

In the first in‐person visit, the physiatrist evaluated the patients, considering the need for rehabilitation and also contraindications or precautions for exercise. People were not candidate to the rehabilitation program if they had potential acute complications, or if they presented conditions preventing them to practice exercise safely,^18^, ^19^ including: hematologic abnormalities (e.g., low platelets, hematocrit and hemoglobin levels, and neutrophil counts), musculoskeletal pain with the suspect of bone metastasis to be diagnosed, cardiovascular disorders (e.g., chest pain, elevated resting heart rates, elevated blood pressure, irregular heartbeats, etc.) or pulmonary disorders (e.g., severe difficulty breathing, coughing/wheezing), or neurological disorders (e.g., decline in cognitive status, dizziness/lightheadedness, disorientation, blurred vision, and increased postural instability). Patients with bone metastases were evaluated for spinal stability with Spinal Instability Neoplastic Score and with Mirels' score for long bones metastases.^20^, ^21^

In the first in‐person visit, the physiatrist and the patient shared the objectives of the rehabilitation program, and the physiotherapist scheduled the appointment for the rehabilitation session.

Supervised rehabilitation sessions could be done either face‐to‐face or in telerehabilitation, in a mixed program set up by the physiotherapist and the patient. When the session was not in‐person, a telemedicine platform was used (C4C Meetings) which guarantees privacy protection according to European Union regulation. A link to participate in the virtual session on the platform via computer, smartphone, or tablet was emailed to each patient.

The rehabilitation program contents included personalized indications regarding exercises (aerobic exercise at moderate intensity, resistance training, flexibility and balance exercises, according to International Guidelines^19^), strategies that could simplify home management or activities of daily living and help to improve QoL, and breathing exercise training (focused on airways clearance or to increase lung capacity), when necessary.

The rehabilitation program included one session per week for 10 weeks, supervised by the physiotherapist (60 min if the session was in‐person, 30 min if the session was delivered with remote care, in videoconference).

Moreover, the patients received via email a tailored list of exercises to be carried out at home, independently or with caregiver support, with clear descriptions and pictures. There were also videos in which the physiotherapist explained the correct sequence of movements and showed how to perform the exercises. The home‐based exercise sessions were scheduled twice a week for 10 weeks. During the program, the physiotherapist enhanced the importance to maintain self‐managed exercise in home‐based session.

### 2.3 Data collection assessment and timeline

We collected general information on patients' sociodemographic and clinical characteristics from their computerized medical records. Patients were assessed at baseline (T0—before starting the rehabilitation program) by a physiotherapist expert in cancer rehabilitation; at the end of the program (T1—3 months after baseline), patients were reassessed by a physiotherapist who had not provided the rehabilitation intervention. The interviews on patients' level of satisfaction and perceived usefulness of the program were also carried out by a physiotherapist who did not provide the intervention. Adverse events were tracked by physiotherapist during face‐to‐face sessions, or to T1 by asking the patient directly (Table 2).

**TABLE 2:** Data collection and assessments at baseline and after rehabilitation program.

	T0‐baseline	T1‐after rehabilitation program
Sociodemographic data (age, sex, household composition, presence of caregiver, work condition, education)	X	
Clinical data (cancer diagnosis and stage, healthcare pathway, use of aids and orthoses, use of pain‐relieving drugs and ongoing cancer therapy)	X	X
Functional outcomes (FSS, EORTC QLQ‐C30, IPAQ‐SF, 30CST)	X	X
Data on rehabilitation program: number of sessions delivered vs number scheduled at baseline; modality (in‐person or telerehabilitation); number of home‐based sessions performed by patient vs number scheduled at baseline; reasons for non‐adherence		X
Patient satisfaction and perceived usefulness		X
Adverse events (fractures, falls and pain exacerbation date of exercise)		X
Reasons for leaving the study		X

At the end of the study, the physiotherapist who delivered the mixed in‐person/telerehabilitation program (AP) was interviewed by a researcher of the study group (MD) to evaluate her satisfaction with the new model of care, its perceived usefulness, advantages, and any criticisms (Appendix S1).

### 2.4 Outcomes

#### 2.4.1 Fatigue

The Fatigue Severity Scale (FSS) is a short questionnaire including nine items that rate the severity of fatigue during the activities performed the previous week on a scale from one to seven. A score ≥36 indicates patients suffering from substantial fatigue who may need further evaluation by a physician.^22^ The FSS has been used to assess fatigue also in the cancer population.^23^ The reliability of FSS is acceptable, with a patients Cronbach's alpha of 0.94, controls Cronbach's alpha of 0.88, and a measurement error of 4.7. Its validity is supported by the significant correlation between the FSS and the EORTC Fatigue subscale (Rs = 0.73 in patients and 0.61 in controls, *p* < 0.001 in both cases).^23^

#### 2.4.2 Quality of life

The European Organization for Research and Treatment of Cancer Quality of Life Questionnaire‐C30 (EORTC QLQ‐C30) is composed of 30 items, including multi‐item scales and single‐item measures. It contains three subscales (functional, symptoms, and global health status), with a score ranging from 0 to 100 for each subscale. Higher scores on the functional scale and on the global health/QoL scale indicate a high/healthy level of functioning and QoL, while a higher score on the symptoms scale indicates a higher level of symptom burden.^24^, ^25^ The EORTC QLQ‐C30 is generally considered to have good reliability, as evidenced by high internal consistency (Cronbach's alpha typically above 0.70) and strong test–retest reliability (ICC typically above 0.70). This scale have also a strong evidence supporting its content, construct, and criterion validity. These metrics support the use of the EORTC QLQ‐C30 as a consistent, stable and well‐validated measure of QoL in cancer patients.^25^, ^26^

#### 2.4.3 Physical activity level

The International Physical Activity Questionnaire—Short Form (IPAQ‐SF) addresses the number of days and the time the patient spent performing PA (moderate/vigorous intensity activities and walking of at least 10‐min duration) in the week before the assessment. It also includes time spent sitting on weekdays. The score, expressed in metabolic equivalent (MET), classifies the level of patients' PA as low (<600 MET), moderate (from 600 to 3000 MET), or high (≥3000 MET).^27^, ^28^ The short version of IPAQ does not have acceptable consistency but remains feasible to administer and handy to combine with other questionnaires.^28^

#### 2.4.4 Lower limb strength

In the 30 s Chair Stand Test (30CST), the physiotherapist asks the participant seated on a chair to stand up from the chair with arms crossed as many times as possible within 30 s. The participant is instructed to fully sit between each stand.^29^ The 30CST is valid, compared to weight adjusted leg press performance for all participants (*r* = 0.77), and reliable, test–retest *r* = 0.89. It can be used as a measure for assessing lower extremity strength and functional capacity across various populations. Its strong correlation with other functional assessments and consistent reliability make it a valuable tool in clinical practice, research, and rehabilitation settings.^29^

### 2.5 Statistical analysis

There was no sample size calculation, but all consecutive patients meeting the inclusion criteria were asked to participate prospectively.

Clinical and demographic data are expressed in terms of frequency and percentage for categorical variables and as mean ± standard deviation for numerical variables.

At the end of the study, we calculated the percentages of:

1. Patients who gave informed consent out of the total eligible (Accrual)
2. Dropouts (Attrition)
3. Sessions carried out compared to sessions scheduled, for supervised (in‐person or via telerehabilitation) and home‐based, respectively. Adherence to the program was defined “good” if 75% of scheduled sessions were carried out. There is little consensus between studies on how adherence should be defined, but we focused on attendance and considered “good” a participation in >75% of all exercise session, like other author in previous studies.^30^, ^31^, ^32^, ^33^

Attrition and adherence were synthesized by percentage accompanied by 95% Clopper–Pearson bilateral confidence intervals (CI); causes of non‐adherence, safety, exit reasons, and patients' user experience are summarized descriptively in tables/lists.

Main clinical scores are summarized as T1–T0 delta, accompanied by a 95% CI; any significant change between the two time points was tested by one‐sample *t*‐test on the delta. Performed tests were considered statistically significant if the *p*‐values were <0.05. Statistical analysis was performed using R 4.3.0 R Core Team [2023].^34^

## 3 RESULTS

**FIGURE 1:**
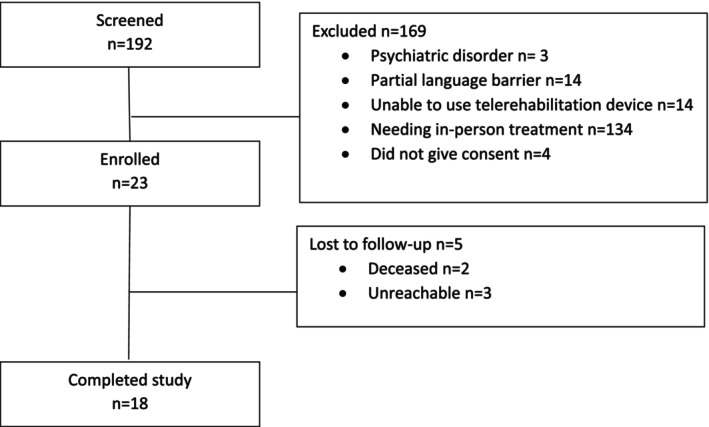
Flowchart of study participant.

In the TRACE study, of the 192 patients screened, 23 were enrolled. Eighteen patients completed the study and underwent the final assessment (Figure 1).

The enrolled patients were evenly distributed between females (47.8%) and males (52.2%), with a mean age just over 60 years and an education level of 13 years (high school diploma). The majority of patients lived with their caregivers (78.3%) and were retired (52.2%).

Concerning the type of diagnosis, 52.2% had a solid tumor (breast cancer was the most frequent, 17.4%), while the other 47.8% had a diagnosis of hematologic cancer. Most of the patients were outpatients referred by the Oncology and the Hematology Departments (87%) (Table 3).

**TABLE 3:** Sociodemographic and clinical characteristics.

Variables	*N* = 23	%
Sex		
F	11	47.8
M	12	52.2
Age		
Mean (SD)	60.35 (11.04)	–
Years of education		
Mean (SD)	13.04 (4.59)	–
Household		
Living alone	5	21.7
Living with caregiver	18	78.3
Occupation		
Employed	10	43.5
Unemployed	1	4.3
Retired	12	52.2
Diagnosis		
Hematologic cancer	11	47.75
Multiple myeloma Lymphoma Leukemia Plasmacytoma Idiopathic myelofibrosis	5 3 1 1 1	21.7 13 4.35 4.35 4.35
Solid cancer	12	52.25
Breast Lung Kidney Abdominal liposarcoma Colon Head–Neck	4 3 2 1 1 1	17.4 13.0 8.8 4.35 4.35 4.35
Healthcare pathway		
Outpatient	20	87
Inpatient	3	13

As regards the clinical data, no changes in percentages were noted between T0 and T1 in the participants who used aids and orthoses, who took painkillers, or in the cancer treatments administered (Table 4).

**TABLE 4:** Clinical data.

Variables	T0 *n* (%) *N* = 23	T1 *n* (%) *N* = 18
Aids and orthosis		
Yes	10 (43.5%)	9 (50%)
No	13 (56.5%)	9 (50%)
Daily pain medication		
Yes	8 (34.8%)	7 (38.9%)
No	15 (65.2%)	11 (61.1%)
Painkillers only when needed		
Yes	10 (43.5%)	8 (44.4%)
No	13 (56.5%)	10 (55.6%)
Cancer treatment		
Chemotherapy	11 (47.8%)	8 (44.4%)
Hormonal therapy	1 (4.3%)	1 (5.6%)
Chemotherapy + hormone therapy	1 (4.3%)	1 (5.6%)
Tumor growth inhibitors	0	1 (5.6%)
No active treatment	10 (43.6%)	7 (38.8%)

### 3.1 Characteristics of intervention

Most of the patients (*n* = 20, 87%) received both in‐person physiotherapy sessions and telerehabilitation sessions, while three patients (13%) exclusively had in‐person sessions. The three patients that had in‐person sessions only, corresponded to inclusion criteria at baseline assessment, but their needs changed during the rehabilitation program: one needed also manual therapy and she was very able to follow exercise program without physiotherapist supervision, so the physiotherapist decided to focus on manual therapy in the in‐person sessions; the other two patients had a fast clinical worsening before telerehabilitation sessions due to brain metastasis.

Regarding the type of intervention, the 20 patients that underwent the mixed treatment intervention did an average of 60% in‐person sessions and 40% telerehabilitation sessions.

In this study, 15 patients did not require the presence of a caregiver during the telerehabilitation sessions (65.2%), while the remaining 34.8% did.

Considering the total length of the rehabilitation intervention, 13 patients were candidated to a short program (<10 sessions), 9 patients to a program of from 10 to 15 sessions, and only 1 person had a long treatment (>15 sessions). The average duration of in‐person sessions was 57.6 min (SD 4.6 min), and 29.3 min (SD 2.7 min) for telerehabilitation. Sessions were scheduled approximately once a week and could be customized according to the patient's needs.

The contents of the rehabilitation treatment depended on the desired goals of each patient (some patients had more than one goal). Goals included muscle strengthening 33.3%, therapeutic education to create an exercise program and manage symptoms (e.g., fatigue) 33.3%, weaning from aids and orthoses 5.9%, assessment and prescription of aids 5.9%, fatigue management and effort reconditioning 7.8%, range of motion recovery 3.9%, greater independence in basic activities of daily living 5.9%, and manual treatment of surgical scar 3.9%.

### 3.2 Feasibility data

Accrual was determined on the 23 patients of the 27 eligible who signed the participation consent form (85%).

Attrition, defined as the percentage of patients who did not complete the study for any reason, was 5 out of 23 patients, equal to 21% (95% CI: 7%–43%).

Ninety‐one percent of the patients had good adherence to supervised sessions (at least 75% of the prescribed sessions completed). The same number demonstrated good adherence to unsupervised home‐based sessions (Table 5).

**TABLE 5:** Adherence and reasons for non‐adherence.

Adherence to sessions supervised by physiotherapist	21 out of 23 (91%; 95 CI: 71%–98%)
Adherence to unsupervised home‐based sessions	21 out of 23 (91%; 95 CI: 71%–98%)
Reason for non‐adherence to sessions supervised by physiotherapist	17 sessions were not completed (7.1%): 9 rescheduled due to health condition (52.9%) 5 for work‐related reasons (29.4%) 2 due to patient difficulty in scheduling the session into daily routine (11.8%) 1 due to a connectivity problem (5.9%).
Reason for non‐adherence to home exercise sessions	72 sessions were not completed (5.8%): 50 of them not performed due to health condition (69.4%) 16 due to lack of motivation (22.2%) 6 due to patient difficulty in scheduling the session into daily routine (8.3%)

Regarding the safety of the intervention, of the 18 patients who completed the study, we registered a total of 5 adverse events: 2 instances of falls (40%), without severe consequences (no fractures nor access to the emergency department), 2 exacerbations of exercise‐induced pain (40%), reduced by rest or shorter exercise sessions, and 1 fracture of the 9th rib during a movement at home (20%). The rib fracture determined an interruption in home‐based exercise sessions.

### 3.3 Clinical outcomes

Table 6 summarizes assessment scores at T0 and T1.

**TABLE 6:** Evaluation scales.

Scales	T0 mean (SD) *N* = 23	T1 mean (SD) *N* = 18
FSS	44.57 (16.01)	38.72 (16.35)
EORTC QLQ‐C30		
Global health status	47.41 (19.58)	56.91 (24.98)
Functional scale	58.45 (23.15)	70.86 (19.65)
Symptoms	33.78 (19.03)	24.07 (15.16)
IPAQ‐SF	1192.04 (1942.74)	3021.50 (5182.90)
30 CST	6.70 (2.58)	8.47 (3.45)
	T0 *n* (%)	T1 *n* (%)
Low (<600 MET)	11 (47.8%)	2 (11.1%)
Moderate (600–3000 MET)	11 (47.8%)	13 (72.2%)
High (>3000 MET)	1 (4.4%)	3 (16.7%)

#### 3.3.1 Fatigue

The mean score of FSS decreased between T0 and T1 (−6.7, 95% CI: −13–−0.437).

Considering the cutoff score of 36 for FSS (≥36 = severe fatigue), 72.2% of the 18 patients who completed the study were classified as having severe fatigue (FSS ≥36) at the initial assessment, whereas only half of them fell into this category at the end of the program.

#### 3.3.2 Quality of life

Looking at EORTC QLQ‐C30 subscales, an increase in mean score was registered between T0 and T1 for global health subscore (delta = +14.3, 95% CI: 6.233–22.509, *p* = 0.002) and functional scale subscore (delta = +13.7, 95% CI: 6.332–21.076, *p* = 0.001), while a decrease was registered for mean subscore of symptoms (delta = −11.8, 95% CI: −18.796–−4.851, *p* = 0.002).

#### 3.3.3 Physical activity level

The IPAQ‐SF mean score increased from 1296.8 MET at T0 to 3021.5 MET at T1. These values refer to physical activity performed by patients in the last week before assessment, according to IPAQ instructions.

At T0, patients were mostly in the low (44.4%) and moderate (50%) intensity categories of PA, while only one person had a high level of PA (5.6%). At the end of the program, most patients^13^ had a moderate level of PA (72.2%) and three a high‐intensity level (16.7%), with only two maintaining a low level of PA (11.1%).

#### 3.3.4 Lower limb strength

The mean score at the 30CST increased between T0 and T1 (delta = +1.5, 95% CI: 6.332–21.076, *p* = 0.002).

Considering the score changes between T0 and T1, an improvement was observed in 12 patients (70.6%), a worsening in 3 patients (17.6%), and an unchanged score for 2 patients (11.8%). It was not possible to evaluate 1 additional patient due to connectivity issues during the T1 assessment.

#### 3.3.5 Patient‐perceived satisfaction and usefulness

Most of the patients stated that they were very satisfied with the program (83.3%), that telerehabilitation was very useful (72.2%), and that there were no critical issues (61.1%). Table 7 shows the difficulties and advantages reported by patients concerning the telerehabilitation sessions.

**TABLE 7:** Difficulties and advantages reported by patients after telerehabilitation sessions.

Challenges encountered by patients during the telerehabilitation sessions No challenges *n* = 12 (66.7%) Few challenges *n* = 4 (22.1%) Several challenges *n* = 1 (5.6%) Many difficulties *n* = 1 (5.6%)
Main difficulties reported by patients about the telerehabilitation sessions Connectivity issues *n* = 6 Lack of physical contact between the physiotherapist and the patient *n* = 3 Difficulties in understanding the exercises to be performed *n* = 2
Advantages of telerehabilitation highlighted by patients Convenience of the service as it avoided having to travel to the hospital *n* = 3

### 3.4 Semi‐structured interview with the physiotherapist

The main results of the interview with the physiotherapist are reported in Table 8, while Appendix S1 contains the entire interview.

**TABLE 8:** Main results of the physiotherapist interview.

Advantages for patients	Disadvantages for patients
Rehabilitation safely at home during the pandemic	Occasional technical difficulties with the IT platform
No need of transport	Difficulties in connecting to the Wi‐Fi network
No architectural barriers	Some patients need in‐person sessions to achieve certain rehabilitation goals (e.g., prescribe and verify aids and orthosis or use of more “traditional” manual physiotherapy techniques)
Early and safe rehabilitation of clinically fragile patients	
Patients' satisfaction	

Advantages for work organization	Disadvantages for work organization
Greater flexibility in scheduling	No contact with patients
Duration of telerehabilitation session was shorter than in‐person session (20/30 min vs 30/60 min), allowing to treat a greater number of patients on the same working day	Need to learn how to use platform for videocalls
Remote treatment allowed direct entry into patients' homes	The physiotherapist must have some particular skills to successfully conduct a telerehabilitation program such as: a good relational skill, ability to create a therapeutic alliance with the caregiver/family and the ability to propose and implement safe rehabilitation exercises to be performed within domestic spaces, with tools available at patients' home
It made monitoring patients for a longer period possible	
With a little training, the physiotherapist could acquire specific skills for using IT platforms for video calls and creating tailored exercise programs to be sent by email	

Conducting both in‐person and telerehabilitation sessions, she noticed that the telerehabilitation sessions were useful and helpful to patients, and they had been positive in terms of work organization, but she also noticed some challenges or critical issues.

She made two suggestions for the future: extending a mixed telemedicine/in‐person program to other rehabilitation‐relevant pathologies and organizing a physiotherapist home assessment before starting the telerehabilitation intervention.

## 4 DISCUSSION

The main objective of this study was to determine the feasibility of a mixed program of telerehabilitation for cancer patients during the COVID‐19 pandemic, and this was the novelty of TRACE 2020 rehabilitation approach. The data show that 85% of patients were offered a mixed intervention modality with both face‐to‐face and telerehabilitation sessions; given the composition of the intervention, on average, 40% of the sessions were carried out in telerehabilitation.

Analyzing adherence to the sessions with the physiotherapist, almost all patients (91%) attended at least 75% of the planned sessions. This promising datapoint can be linked to the great flexibility offered by a mixed intervention modality that meets patients' needs, thereby increasing their motivation to participate and thus adherence.

Home sessions, carried out independently by the patient, also showed very good adherence: 91% of patients attended at least 75% of the planned sessions. The key element for enhancing adherence to the intervention may be to develop the most adequate home based‐exercise program for each patient and to monitor it over time, as stated by Galliano Castillo, who reported very good adherence (94%) for home‐based sessions in breast cancer patients.^8^

The secondary objective was to evaluate the impact of telerehabilitation on functional outcomes: from T0 to T1, patients showed a decrease in perceived fatigue, an improvement both in global health status and functional score of the EORTC QLQ‐C30 and a decrease in symptom score. These findings are in accordance with other studies, underlining that telemedicine interventions can help to improve the HRQoL of cancer patients or at least help to prevent it from deteriorating.^9^

Despite the lack of a control group, the trend of the assessment scale scores is in line with the improvements in QoL and fatigue obtained in previous RCTs that tested telerehabilitation versus control.^35^

Regarding physical outcomes, we noticed an improvement in 30CST score for 70.6% of patients. However, the most interesting finding was the considerable increase in the average level of PA, which more than doubled from T0 (1296.8 MET) to T1 (3021.5 MET). Moreover, at the end of the rehabilitation program, most patients had a moderate (72.2%) or high‐intensity (16.7%) level of PA. Progressive resistance exercise, moderate intensity aerobic exercise and home‐based exercise sessions improved PA level. These findings are consistent with the study by Dennet et al., who stated that telerehabilitation improved self‐reported PA levels in cancer patients, especially when sessions were associated with the possibility of maintaining personal contact through phone calls or in‐person sessions.^36^

Lastly, all the patients answered the final questionnaire, revealing high levels of satisfaction (83.3%) and of the perceived usefulness (72.2%) of the telerehabilitation sessions. In line with the previous literature,^8^, ^35^, ^36^, ^37^ both the patients and HCP agreed on the advantages of using telerehabilitation: it eliminates having to travel to a healthcare facility for rehabilitation, it allows practitioners to take care of patients living far away and it saves patients time and the expense of traveling from home to the healthcare facility. The most fragile and clinically deconditioned patients can therefore participate in the telerehabilitation session in their best psychophysical conditions, without the added stress of the journey, which can often be very challenging for them. Furthermore, telerehabilitation allowed high‐risk cancer patients to do rehabilitation during the COVID‐19 pandemic despite the general advice to minimize their visits to a hospital.

Another reported strength of telerehabilitation is its flexibility and ease of use. Patients had little need for caregiver support, which was requested largely only during the first session so as to become familiar with the technology. In two thirds of cases, no difficulties were reported, confirming that telerehabilitation is generally perceived as a positive experience.^36^, ^38^, ^39^ Shorter session duration (20–30 min, vs. 30–60 min of in‐person rehabilitation) allowed HCP to monitor the activities carried out at home of a greater number of patients per day, for a longer period of time.

As regards the difficulties that emerged during the telerehabilitation sessions, there were issues in starting the video call, mainly for older patients, or internet access problems; both patients and HCP emphasized the loss of physical contact and that there were some problems in understanding how to perform an exercise. This could be the reason why a mixed intervention is to be preferred, whenever possible, as also suggested by other studies.^36^

Home‐based interventions also have some disadvantages, and key elements must be considered: a potential lack of exercise equipment, inadequate space for exercising at home, limited access to technology or low technology literacy, inability to assess patients in‐person, and potential limitations concerning safety, namely in‐person monitoring of exercise response (i.e., heart rate, blood pressure) and hands‐on assistance with exercise or movement technique.^40^

To improve the use of telerehabilitation in the future, we think that it could be useful for the physiotherapist to perform a preliminary visit to the patient's house, if there are doubt about home environment and safety, after asking patients and their caregivers information. Other authors performed an initial physiotherapist assessment of patients' home environment to evaluate the setting, its safety and the items already available in the patient's home to establish personalized exercises and adapted aerobic exercise.^7^

The three patients that had in‐person sessions only (one because of the need of manual therapy and two for clinical worsening before telerehabilitation sessions due to brain metastasis) underline the need to more carefully define the characteristics of the ideal patient to whom telerehabilitation should be proposed, as also suggested in the literature.^41^ In 2021, telemedicine was suggested as an invaluable way to continue cancer rehabilitation services, even if some concerns arose for patients with specific characteristics such as worsening of pain or neurologic deficits, risk of bone lesions, suspected spinal involvement, new muscle‐skeletal complaint, chemotherapy‐induced peripheral neuropathy affecting gait, and so on.^21^ Telerehabilitation is now considered a branch of medicine, but some cancer patients require a very careful evaluation before starting any program, which includes remote assessment and treatment.^21^, ^42^ Our results suggest that advanced stage cancer patients should be excluded as well as patients who have reduced cognitive performance due to brain cancer or metastasis.

### 4.1 Strengths and limitations of the study

One limitation of this study is that it included a small heterogeneous nonrandomized sample of patients; the data on functional outcomes therefore cannot be compared to a control group, and the results on functional evaluations cannot be generalized. However, the lack of a control group was a specific choice of the research group; during the pandemic, we chose to offer access to a mixed program that included telerehabilitation to all eligible cancer patients, for ethical and emergency reasons.

It was not possible to make a cost assessment in our context, as another study has,^36^ but it would be an interesting aspect to investigate in future research. However, telerehabilitation may have advantages in terms of cost; the shorter duration of each session means that HCP can offer rehabilitation to a larger number of patients in the same amount of time.

To our knowledge, this is the first study to evaluate the feasibility of a cancer telerehabilitation intervention in an Italian public healthcare setting for a large cancer population, not only patients with breast cancer.^13^ In a period when health services were required to explore new areas and strategies in order to guarantee the care of more fragile categories of patients, this study allowed us to investigate the effects of introducing telerehabilitation in our context in terms of the impacts on clinical and functional outcomes and to acquire feedback from HCP and patients.

Moreover, looking at international context, no previous studies investigated a mixed in‐person and telerehabilitation program for cancer patients; the results of our study are encouraging and contribute to enhance the importance to explore in future studies a mixed‐mode delivery method of rehabilitation intervention in cancer care on larger samples of participants.

## 5 CONCLUSIONS

This study found that a mixed in‐person/telerehabilitation intervention is feasible, accepted by patients, it can facilitate programs having a positive effect on cancer patients' fatigue, QoL and level of PA, and it makes patient care more flexible. Further research is required to identify the characteristics of target populations for telerehabilitation and to confirm the benefits of this kind of intervention so that it can be integrated into standard care.

## PRECIS

Covid‐19 pandemic, having required rapid changes in healthcare provision, has underlined the usefulness of telemedicine as an innovative approach both for patients and healthcare organizations. A mixed rehabilitation program including telerehabilitation is feasible and accepted by cancer patients and healthcare professionals; it may have a positive effect on cancer patients’ fatigue, QoL and level of PA and makes patient care more flexible.

## AUTHOR CONTRIBUTIONS

**Monica Denti:** Conceptualization (lead); data curation (equal); formal analysis (equal); investigation (lead); methodology (lead); visualization (equal); writing – original draft (equal); writing – review and editing (equal). **Alessia Pecorari:** Conceptualization (supporting); data curation (lead); formal analysis (equal); investigation (equal); methodology (equal); resources (equal); writing – original draft (equal); writing – review and editing (equal). **Monia A. Accogli:** Conceptualization (equal); data curation (equal); formal analysis (equal); investigation (equal); methodology (equal); validation (equal); writing – original draft (equal); writing – review and editing (equal). **Stefania Costi:** Data curation (supporting); writing – original draft (supporting); writing – review and editing (supporting). **Carlotta Mainini:** Data curation (supporting); writing – original draft (supporting); writing – review and editing (supporting). **Martina Pellegrini:** Data curation (supporting); writing – original draft (equal); writing – review and editing (equal). **Chiara Boni:** Data curation (supporting). **Bressi Barbara:** Data curation (equal); writing – original draft (equal); writing – review and editing (equal). **Luca Braglia:** Software (lead). **Stefania Fugazzaro:** Conceptualization (lead); data curation (lead); formal analysis (lead); investigation (lead); methodology (lead); project administration (lead); supervision (lead); validation (lead); visualization (lead); writing – original draft (lead); writing – review and editing (lead).

## FUNDING INFORMATION

This work was supported by Azienda USL‐IRCCS of Reggio Emilia and partially supported by the Italian Ministry of Health—Ricerca Corrente Annual Program 2025.

## CONFLICT OF INTEREST STATEMENT

The authors declare no conflicts of interest.

## Supporting information


Appendix S1.

## Data Availability

The dataset generated and analyzed in the current study was managed by the Information and Technologies Service (STIT) of the Azienda USL–IRCCS of Reggio Emilia in order to protect patient privacy. The data that support the findings of this study are available from the corresponding author upon reasonable request.
