# Supplementary material for: Facing the COVID‐19 pandemic: An Italian feasibility study of a mixed in‐person/telerehabilitation intervention for cancer patients
Source: Cancer Med. 2024 Aug 2;13(15):e70022. doi: 10.1002/cam4.70022 (PMC11296954; doi:10.1002/cam4.70022)
Supplement: Supplementary file 1 — Appendix S1. [file CAM4-13-e70022-s001.docx]

**APPENDIX 1 - PHYSIOTHERAPIST QUESTIONNAIRE**

PERCEPTION OF USEFULNESS AND PATIENT SATISFACTION

- **Do you think the telerehabilitation intervention was useful for patients? Why?**

Yes, I think it was useful; onco-haematological patients could remain safely at home during the pandemic and carry out rehabilitation without the risk of contracting the COVID-19 infection.

The time of rehabilitation via video call was more flexible, as it was not necessary for a caregiver/ambulance to transport the patient from his/her home to the hospital.

If environmental barriers were present, it was not necessary to overcome them.

It was possible to conduct the rehabilitation of patients living far away.

It was possible to carry out early and safe rehabilitation of very fragile patients from a clinical point of view (for example, patients who had just been discharged from hospital after a bone marrow transplant, who were highly immunosuppressed).

- **Do you think the patients were satisfied with the telerehabilitation intervention method? Why?**

Generally speaking, I think the patients were satisfied with a new, innovative model particularly useful during the pandemic.

STRENGTHS AND ISSUES OF THE TELEREHABILITATION INTERVENTION

- **What were the positive aspects/strengths in carrying out the telerehabilitation sessions?**

I think the positive aspects were:

- Ease of using the IT platform for the video call

- Sharing brochures, personalized exercise programmes accompanied by photos, explanations and videos of the correct execution during the video call.

- Greater flexibility in hours.

- Absence of the need to involve a caregiver to transport the patient to hospital or use an ambulance.

- Possibility of taking care of patients living far away, saving the patient travel time and expense from home to hospital.

- Furthermore, this innovative modality allowed the most fragile and clinically deconditioned patients to access the telerehabilitation session in the best psychophysical conditions, without any home-hospital transport-related stressed; transport is often very demanding and difficult for the patient and the caregiver.

- The remote treatment method allowed me to enter "live" into patients' homes, to understand their family dynamics more deeply and to suggest useful strategies for functional recovery and how to simplify home management.

- Ability to monitor the proposed exercises for a longer period of time

-Possibility to recommend how to increase the difficulty of exercise execution in the medium-long term.

- Possibility of using the telerehabilitation tool even after the pandemic period not only for onco-haematological patients but also for patients with other pathologies that require rehabilitation.

- **What critical issues did you encounter when carrying out the telerehabilitation sessions?**

I sometimes had technical difficulties with the platform used for the video call and sometimes difficulties in connecting with the company Wi-Fi network, which were resolved with the intervention of the IT technician.

To achieve certain rehabilitation goals, a face-to-face session was more suitable and effective than a video call, given the need for physical contact and the need to use more "traditional" manual physiotherapy techniques.

Some older patients reported IT difficulties and some problems in managing technology (use of e-mail, ability to authorize microphone and camera to start the video call); these difficulties were almost always overcome thanks to the support of a caregiver.

PROFESSIONAL COMPETENCE

- **What skills did you acquire or have to learn to carry out the telerehabilitation intervention?**

I had to acquire specific skills for the use of an application provided by the company, which allows the drafting of personalized rehabilitation programmes that can be sent via email or in paper format through a distance learning course (FAD). I learned how to use a specific platform that meets security and privacy criteria for the use of virtual rooms for telerehabilitation activities.

- **Which specific physiotherapist skills, instead, were not used in the telerehabilitation intervention?"**

Manual techniques in general that involve physical contact, and assessment of aids/orthotics.

INTEGRATION OF TELEREHABILITATION INTO PHYSIOTHERAPISTS’ DAILY WORK

- **To make the intervention transferable to other professionals in the field of telerehabilitation for oncology patients, what skills do physiotherapists require?**

Specific skills for using an application provided by the company to create personalized rehabilitation programmes are required. Proficiency in using a specific platform, which meets security and privacy criteria for the use of virtual rooms in telerehabilitation activities, is essential. Good interpersonal skills, even at a distance, are necessary. Ability to establish a therapeutic alliance with the caregiver/family when needed. Capability to propose and implement safe rehabilitation exercises to be performed at home, using readily available everyday objects.

- **How did the use of telerehabilitation impact the daily organization of your work?**

I find it was very useful from an organizational perspective, primarily for greater flexibility in scheduling video calls. For example, when patients are unable to travel to the hospital independently, it is necessary to make arrangements with caregivers, ambulances, or volunteer organizations for patient transport to hospitals. With telerehabilitation, instead, finding the right moment for an appointment is easier. Moreover, during the pandemic there was a shortage of available volunteer personnel, and transport to hospital was sometimes difficult.

Often, the duration of the telerehabilitation sessions varied from 20 to 30 minutes, while in-person sessions can vary from 30 to 60 minutes. From an organizational standpoint, telerehabilitation has allowed me to clinically follow a greater number of patients within the same working day and to have them in care for a longer period. This has enabled me to conduct remote monitoring of physical activities to be performed at home for an extended period of time.
